# Interventions to Support International Migrant Women's Reproductive Health in Western-Receiving Countries: A Systematic Review and Meta-Analysis

**DOI:** 10.1089/heq.2020.0115

**Published:** 2021-05-25

**Authors:** Kara Redden, Jessica Safarian, Claudia Schoenborn, Clare Shortall, Anita J. Gagnon

**Affiliations:** ^1^ Ingram School of Nursing, McGill University, Montréal, Canada.; ^2^ Research Centre in Health Policies and Health Systems, School of Public Health, Université Libre de Bruxelles, Brussels, Belgium.; ^3^ Doctors of the World UK, Part of the Médecins du Monde Network, London, United Kingdom.; ^4^ Reproductive Outcomes and Migration (ROAM) Collaboration.

**Keywords:** emigrants and immigrants, transients and migrants, reproductive health, women, culturally competent care

## Abstract

**Purpose:** The reproductive health outcomes of international migrant women differ in comparison with receiving-country-born women, depending on country of birth and immigrant status. Effective interventions to support the reproductive health of international migrant women are not well known.

**Methods:** We conducted a systematic review and meta-analysis of studies between 2010 and 2017 evaluating interventions directly or indirectly affecting the reproductive health (as defined by the World Health Organization) of international migrant women in Western-receiving countries.

**Results:** Sixteen studies representing 5080 migrants were identified. Interventions consisted of linguistically (e.g., translated brochures) or culturally adapted (e.g., cultural narratives) routine care or new interventions. Meta-analysis showed that interventions increased rates of preventive reproductive health activities, including mammography, condom use, and Pap test completion, by almost 18% (95% confidence interval 7.61–28.3) compared with usual care or interventions not adapted to migrant women.

**Conclusion:** Culturally and linguistically adapted care practices congruent with target populations of international migrant women are effective in improving their reproductive health outcomes, particularly their participation in preventative reproductive health activities.

Women constitute nearly half of the 244 million migrants who have resettled worldwide and up to 52% in some Western industrialized nations.^1^ As such, an international call for research and action was made to address the health needs of international migrant women.^2,3^ The course of migration (e.g., pre, during, and post) for women often involves experiences that can negatively impact their reproductive health, defined by the World Health Organization (WHO) as “reproductive health processes, functions and systems at all stages of life.”^4^ These experiences can include poor access to reproductive health services, gender-based/sexual violence, and granting of sexual favors for security or food, among others.^2,5,6^ They are compounded by additional challenges in access to reproductive health care services in Western-receiving countries, such as language and cultural barriers, immigration policies delaying residency status, access to health care insurance, administrative barriers including financial burden, and health system barriers including gender-based inequity for health care provider preference and discrimination.^2,6–8^

Over the past decade, reproductive health disparities have focused on perinatal health, which have been well-documented between groups of international migrant women and women from Western-receiving countries.^9–25^ Despite migrant subgroup variations, the disparities often include an increased risk of the following: low birth weight and preterm birth,^18,19,21,22,24–26^ inadequate prenatal care,^12^ emergency cesarean birth,^15^ and perinatal death.^17^ These outcomes vary according to region of origin. For example, Southeast Asian women have a higher risk of preterm delivery (odds ratio [OR] 1.41, 95% confidence interval [CI] 1.02–1.96),^18,19^ North African women have a higher risk of perinatal/infant mortality (OR 1.15–1.42),^9^ and North African (OR 1.11, 95% CI 1.03–1.20^15^) and Latin American (OR 1.59, 95% CI 1.13–2.25^15^) women have higher rates of emergency cesarean birth compared with receiving-country women.^16^ Sub-Saharan African women have higher risks for multiple poor perinatal health outcomes (e.g., small-for-gestational age [OR 1.31, 95% CI 1.14–1.50],^20–22^ preterm birth [OR 1.33, 95% CI 1.17–1.50],^18–21,24^ and emergency cesarean birth [OR 1.38, 95% CI 1.06–1.80]).^19,26^ While these worrisome disparities between migrant and Western-born women are widespread, there is also documented evidence of similar or better perinatal outcomes for certain international migrant women. For example, North African women have lower risks of small-for-gestational age births (OR 0.71, 95% CI 0.69–0.73) compared with receiving-country women.^20–22,24^

There is a lack of research on a broader range of reproductive health issues among international migrant women. The scope has generally been limited to perinatal health outcomes and has failed to address other elements of reproductive health such as menopause, sexually transmitted infections, and sexual health behaviors.^10^

Efforts to improve the care of culturally and linguistically diverse migrant populations in regions of the industrialized West have been reported. The European initiative *Migrant Friendly Hospitals*, responding to the changing political and cultural climate of Europe, called for effective interventions to promote the health of migrant and ethnic minority groups.^27^ Culturally and linguistically adapted interventions were recommended to improve health care delivery and health outcomes among migrant and allophone patients. Similar initiatives have since been reported in the United States.^28^ These interventions have not been specific to the reproductive health needs of international migrant women.

Given the vulnerability of international migrant women disparities in their reproductive health outcomes compared with receiving-country women, there is an impetus for practicing professionals to acquire knowledge and tools to effectively support their reproductive health needs, and for health care systems to adapt to these needs. It is also imperative to identify interventions that promote health and preserve protective factors seen in various migrant groups. To our knowledge, little is currently known about the types or effectiveness of interventions used to support international migrant women's reproductive health.

The Reproductive Outcomes and Migration (ROAM) international research collaboration has initiated this investigation to address the following research question: Using systematic review and meta-analyses, what is the effectiveness of interventions directed to international migrant women settling in Western countries, which may directly or indirectly affect their reproductive health? We use the PRISMA reporting style to describe our project.^29^

## Methods

### Eligibility criteria

#### Interventions included

We selected studies published in any language assessing the effectiveness of any intervention offered to international migrant women in Western-receiving countries on aspects of their general health, ultimately impacting their reproductive health or their reproductive health directly. We included those examining general health for two reasons: (1) The number of studies focusing on interventions to benefit reproductive health were few and (2) we suspected that at least some components of interventions effective for the general health of international migrant women might also be effective for their reproductive health outcomes. When interventions were also provided to men or to the couple as a unit, we included the studies if the outcomes for women were reported separately.

#### Interventions excluded

Studies exclusive to men's reproductive health interventions, those that did not report outcomes for a comparison group, and population interventions such as policies were excluded.

#### Population included

International migrant women were identified as those who had crossed international borders and settled in an Organization for Economic Co-operation and Development (OECD) or a European Union (EU)-27 country. Studies were included if the results provided enough information to draw conclusions for international migrant women (i.e., if more than 80% of the study sample were foreign-born women or if outcomes for foreign-born women were presented separately).

#### Population excluded

Studies of populations labeled as second-generation migrants were excluded since those women are not migrants.

#### Outcomes included

Reproductive health, as defined by the WHO, “addresses the reproductive processes, functions and system at all stages of life,” in which “people are able to have a responsible, satisfying and safe sex life and that they have the capability to reproduce and the freedom to decide if, when and how often to do so”.^4^ Studies, therefore, included those addressing reproductive health outcomes directly, such as gestational diabetes and postpartum depression, and indirectly, such as type II diabetes and mental health.^30–33^

### Information sources and search strategy

**FIG. 1.**
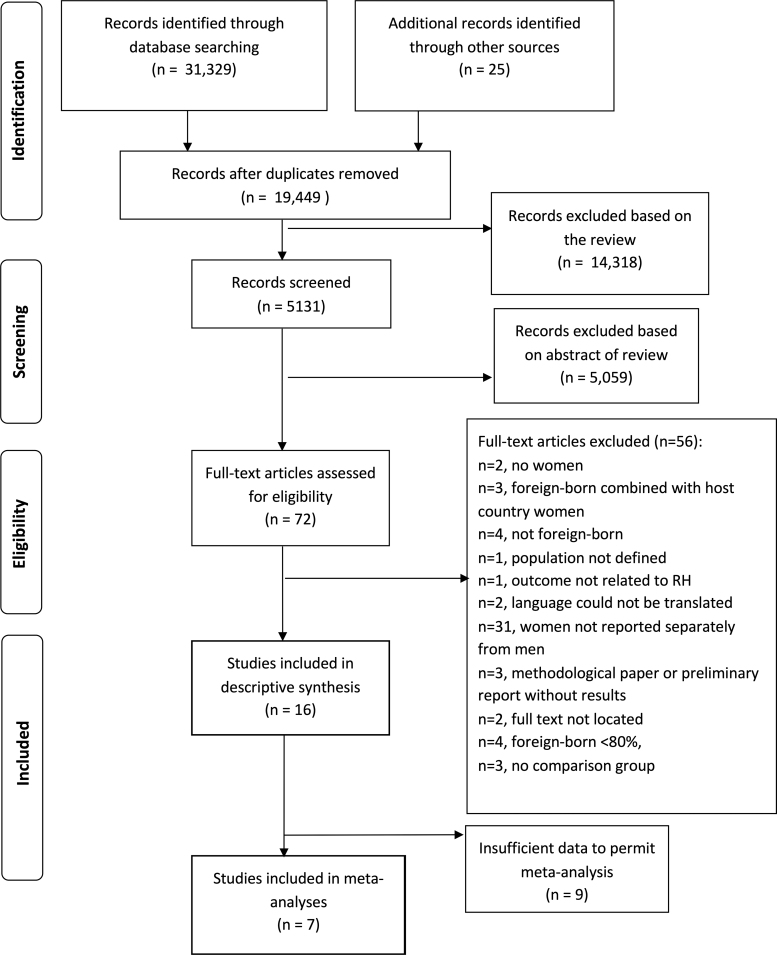
PRISMA reporting diagram. RH, reproductive health.

We identified published reports by searching electronic literature databases from January 1, 2010, to July 30, 2017, using Medline, Embase, PsycINFO, Global Health, Social Work Abstracts, CINAHL, Joanna Briggs Institute, Cochrane Library, Web of Science, and ProQuest Dissertations and Theses, ISRCTN, and gray literature (Supplementary Appendix SA1). We chose January 2010 as our starting date because it immediately follows completion of our previous review.^9^ We extended our search to mid-2017 due to the high number of research result records for review (Fig. 1). The search strategy was developed in collaboration with a McGill University Health Sciences Librarian. Abstracts of intervention studies were scanned for comparator key words (e.g., “difference in,” “compared to,” “between”) and used to focus our search strategy given the large volume of descriptive, noncomparative literature available on international migrant women's reproductive health. This systematic review did not require a formal IRB approval.

### Study selection, data collection process, and data items

Three research assistants and two authors independently assessed the articles for study eligibility. Studies were then evaluated for risk of bias and qualitatively synthesized.

Data extracted from each study included the following: year of the report, receiving country, size of intervention group, size of comparison group, calendar years of data, and migration indicators recommended for use in research of international migrant women (i.e., language fluency, ethnicity, country of origin, length of time in country, and immigrant status^34^). Data extracted separately for the intervention and comparison groups included the following: intervention(s) tested (e.g., brochures, group education, audiovisual media); intervention provider/mechanism (i.e., research staff, community health worker, practicing health care professional, computer); intervention setting (i.e., clinic, home, community, hospital); intervention mode (i.e., group, individual, both); and number and duration of intervention sessions (if applicable).

Outcomes were classified into broad categories. Those directly associated with reproductive health were placed into categories, including nonperinatal reproductive health outcomes (e.g., reproductive cancers, sexually transmitted infections, and sexual and reproductive health behaviors such as condom use); perinatal/infant outcomes (e.g., maternal health promoting behaviors such as infant feeding/breastfeeding knowledge and behaviors); and psychosocial outcomes such as postpartum depression. Outcomes indirectly associated with reproductive health included health-promoting behaviors (e.g., physical activity, dietary habits) and psychosocial outcomes such as depression knowledge.

### Risk of bias in individual studies

Risk of bias was assessed for experimental studies using the Cochrane Collaboration risk of bias tool.^35^ Quasiexperimental studies were assessed for level of risk of bias using the ROBINS-I tool.^36^ Two authors (K.R. + J.S.) assessed the risk of bias independently for all studies and assigned a low, high, or unclear summary assessment for each. Discrepancies in assessments were discussed and agreed upon before finalizing overall summary assessments. A third author (A.J.G.) confirmed agreement.

### Summary measures and synthesis of results

Studies were described with respect to data extracted and previously defined direct and indirect reproductive health outcome variables. We used reported statistical significance (*p*-values <0.05 or reported to be significant by authors) to classify the effects of the intervention as being better, worse, mixed (migrant subgroups within the same study having dissimilar results), or no different than the comparison group. We analyzed subgroups of studies defined by risk of bias. Studies reporting outcomes using similar measurements were combined for meta-analysis when possible. If studies reported on similar outcomes while using different scales, the lowest common denominator of the scales was used to permit combining results across studies. The effect size of each intervention was determined by calculating the mean difference between intervention and comparison group outcomes. We calculated confidence intervals of the mean difference based on reported standard deviations and sample sizes. Given the heterogeneity of outcomes, we used a random-effects meta-analysis using the inverse-variance method in Review Manager© 5.3.

### Risk-of-publication bias across studies

Articles that met the eligibility criteria and systematic reviews reporting on reproductive health outcomes were hand-searched for additional studies. Efforts were made to contact all authors of eligible studies to identify additional studies. When articles could not be professionally translated, we contacted the authors to request information in English about the study. Authors of abstracts meeting inclusion criteria were contacted to locate full-text articles when we could not locate them. A funnel plot was created, but given the eventual low number of studies available was not informative in assessing publication bias.

## Results

### Study selection

We initially identified 31,354 studies. After eliminating duplicates, 19,449 remained. Abstracts and titles were then reviewed and eliminated if exclusion criteria were met, yielding 72 full-text articles to review (Fig. 1). Fifty-six were found to meet exclusion criteria, leaving 16 studies included in the review. Seven studies reported results permitting meta-analyses.

### Study characteristics

The characteristics and individual results of each of the 16 studies are presented in Table 1. Thirteen randomized-controlled trials and three quasiexperimental studies representing 5080 migrants were included. All 16 studies were written in English, with the majority of studies (88%) having been conducted in the United States. More than half of the studies (56%) had sample sizes of <250 international migrant women. Studies largely included Latina/Hispanic (50%) and Chinese (25%) international migrant women. Of the five recommended migration indicators to be used in research with migrant populations,^34^ ethnicity and country of birth were predominantly used (54% and 45% of studies, respectively). Language fluency was reported in eight studies (50%) and length of time in country was reported in two (13%). No studies used immigration status as an indicator of migration.

**Table 1.** Characteristics and Results of Individual Studies

Low risk of bias (n=3)				
Study	Study characteristics	Description of study intervention	Description of comparison	Results (study intervention vs. comparison)
Koniak-Griffin (2015)^51^	Design: RCT Population: Latinas, 35–64 years old, BMI >25 (*n*=223) Location: United States Setting: Home and community	Spanish lifestyle behavior intervention using *Mujeres Sana y Precavidas* curriculum—a culturally relevant educational program to promote a healthy lifestyle (through diet and physical activity) to reduce cardiovascular disease. Teachings included information on cardiovascular disease, how to achieve personal goals, support behavior changes, and provide guidance on how to overcome barriers to lifestyle behavior change. Visual displays, videos, and role-playing were also included. Patients also received a pedometer to measure physical activity (*n*=111) Development: National Heart, Lung and Blood Institute Provider: *Promotoras* (CHWs) Group vs. Individual: Both Number/duration of sessions: 16 sessions×6 months, 2 h/session	Promotoras led, 6-month safety, disaster preparedness educational program (*n*=112) Development: Not described Provider: Separate team of *promotoras* Group vs. Individual: Both Number/duration of sessions: 16 sessions×6 months,	Positive effect: • Increased mean dietary habits score (2.26 vs. 2.08, *p*<0.001) • Decreased mean waist circumference (99.32 cm vs. 99.77 cm, *p*<0.05) • Increased mean number of steps/physical activity from baseline to 9 months (contrast *t*=2.07, df=201, *p*=0.04). No statistical difference in effect: • Mean BMI (31.96 vs. 32.99, *p*=ns) • Mean weight (171.40 lbs vs. 176.60 lbs, *p*=ns) • Mean cholesterol levels (185.48 mg/dL vs. 189.30 mg/dL, *p*=ns) • Mean fasting blood glucose values (99.31 mg/dL vs. 99.44 mg/dL, *p*=ns)
Thompson (2012)^49^	Design: RCT Population: Spanish-speaking Latinos (*n*=160) Location: United States Setting: Hospital clinic	Intervention: Five Spanish interactive educational modules based on *Bright Futures Guidelines for Health Supervision of Infants, Children and Adolescents.* Guidelines were presented on a touch screen computer based on common beliefs and practices about infant/toddler nutrition and feeding in Latino immigrant populations. Included use of text, audio, and images. (*n*=80) Development: *Bright Futures Guidelines* plus literature used by authors Provider: Touch screen computers Group vs. Individual: Individual Number/duration of sessions: 1 session, 5 modules, 2–8 min/module	No care (*n*=80)	Positive effect: • Increased summed knowledge scores of all domains on infant/toddler nutrition and feeding (90.8% vs. 72.3%, *p*<0.001); o Significant differences consistent between intervention and control for each domain (breastfeeding, formula feeding, milk, juice, and solid foods).
Wingood (2011)^50^	Design: RCT Population: Latina women, 18–35 years old (*n*=252) Location: United States Setting: Community primary care clinic	*AMIGAS*, Spanish HIV sexual risk reduction intervention using culturally relevant themes about ethnic pride, social norms, HIV misconceptions, and role-playing (*n*=125) Development: Latina health educators through focus groups with Latina women Provider: Latina health educators Group vs. Individual: Group Number/duration of sessions: 4 sessions, 2.5 h/session	General health comparison intervention that provided basic HIV information (*n*=127) Development: Field testing with Latina women Provider: Latina health educator Group vs. Individual: Group Number/duration of sessions: 1 session, 2.5 h	Positive effect: • Increased consistent condom use over 90-day period (39.0% vs. 14.3%, aOR 4.87, 95% CI 2.27–10.42)

High risk of bias (n=5)				
Study	Study characteristics	Description of intervention	Description of comparison	Results (study intervention vs. comparison)
Ma (2015)^37^	Design: RCT Population: Vietnamese women aged 21–70 (*n*=1488) Location: United States Setting: Community-based organization	Cervical cancer education using culturally relevant visual aids including pictures of Vietnamese women, patient navigation, communication and doctors available in English and Vietnamese, referral to Pap test sites, and 6-month screening reminders (*n*=816) Development: Vietnamese community leaders and focus groups Provider: Professionally trained community health educators Group vs. Individual: Group Number/duration of sessions: 1 session, duration n/a	Information about general health issues with translated material from federal agencies and community-based organizations (*n*=672) Development: Not described Provider: Not described Group vs. Individual: Not described Number/duration of sessions: Not described	Positive effect: • Higher self-reported rates of Pap test within 12 months (60.1% vs. 1.6%, *p*<0.001); • Higher rates of planning to have Pap test postintervention (*p*=0.002) and at 12-month follow-up (*p*=0.001) (no individual data)
Pitcock (2015)^47^	Design: Quasi-experimental Population: Hispanic women Location: United States Setting: Hospital	Prenatal class educational program to provide information about breastfeeding congruent with cultural beliefs and practices of the target group—using activities, tactile and visual aids, novella. Offered in Spanish (*n*=38) Development: Culturally competent education curriculum **Provider:** Bilingual, trained health educators Group vs. Individual: Group Number/duration of sessions: 1 session×6 h	Usual care—a series of optional childbirth education classes taught in English ( *n*=32) Development: Not described Provider: Not described Group vs. Individual: Group Number/duration of sessions: 8 sessions, unknown duration	Positive effect: • Higher rates of exclusive breastfeeding at discharge (41% vs. 3.1%, *p*=0.001) No statistical difference in effect: • Rates of intent to breastfeed at admission (53.8% vs. 37.5%, *p*=0.08)
Scheinmann (2010)^45^	Design: Quasiexperimental Population: Latina women, >18 (*n*=272) Location: United States Setting: Home	Culturally appropriate educational video on age-appropriate foods and infant feeding practices in English and Spanish. (*n*=143) Development: Professional production team Provider: Trained bilingual research staff Group vs. Individual: Individual Number/duration of sessions: 1 session, 25-min video	No care, did not receive DVD	Positive effect: • Higher percentage of knowing what age to introduce solids (74.4% vs. 56.6%, *p*<0.01); • Greater overall change in knowledge scores related to obesity (OR 1.7, *p*=0.01); • Knowledge of mean age of introduction of solids (5.2 vs. 4.9 months, *p*=0.02); No statistical difference in effect: No difference for mean age of introduction of juices was found between video group and comparison group (*p* value not reported)
van der veen (2014)^42^	Design: Clustered three-group RCT Population: Turkish women, 16–40 years old (*n*=779) Location: Netherlands Setting: Home	Behavioral and cultural therapy intervention using computer modules on Hepatitis B screening, knowledge, social norms, support available in Dutch or Turkish. (*n*=248) Development: Turkish organizations Provider: Computer (via postal invitation from municipal health service) Group vs. Individual: Individual Number/duration of sessions: 1 session, duration n/a	Behavioral therapy only intervention and generic online information from the National Hepatitis Center (*n*=293) Development Turkish organizations and National Hepatitis Center Provider: Computer (via postal invitation from municipal health service) Group vs. Individual: Individual Number/duration of sessions: 1 session, duration n/a	No statistical difference in effect: • Hepatitis B screening rates observed between the three groups (43.9% vs. 43.5% vs. 46.0%, *p*=0.74); • Mean screening intention scores (0.23 vs. 0.23 vs. 0.22, *p*=0.63) Positive subeffect Only women <30 in intervention group had higher screening rates (55% vs. 33% vs. 46.0%, *p*=0.07) and mean screening intention scores (3.62 vs. 3.33 vs. 3.31, *p*=0.07)
Wang (2010)^39^	Design: Quasiexperimental Population: Chinese women, >18 (*n*=134) Location: United States Setting: Community	Chinese education sessions about cervical cancer in relation to life roles of Asian women. Included group discussion, Chinese handouts and videos as well as patient navigation and assistance. (*n*=80) Development: Community leaders Provider: Chinese community health educators Group vs. Individual: Group Number/duration of sessions: 1 session, duration n/a	Health education sessions covered topics on general health and received written material on general health and cancer screening (*n*=54) Development Community leaders Provider: Chinese community health educators Group vs. Individual: Group Number/duration of sessions: 1 session, duration n/a	Positive effect: • Higher rates of identifying risk factors for cervical cancer (HPV infection 89.98% vs. 27.3%, *p*<0.01; multiparity 84.8% vs. 18.2%, *p*<0.001, having sex at a young age 84.8% vs. 63.6%, *p*<0.01) • Higher rates of identifying symptoms of cervical cancer (vaginal discharge 93.4% vs. 84.8%, pelvic pain 88.2% vs. 26.1%, *p*<0.001, pain during intercourse 94.7% vs. 32.6%, *p*<0.001) Higher cervical cancer screening rates (70% vs. 11%, *p*<0.001)

Unclear risk of bias (n=8)				
Study	Study characteristics	Description of study intervention	Description of comparison	Results (study intervention vs. comparison)
Hernandez and Organista (2013)^43^	Design: RCT Population: Latina women aged 18–55 (*n*=142) Location: United States Setting: Community primary care clinic	Fotonovela *Secret Feelings*, a comic book layout with Spanish words and characters with storylines about a depressed middle-age Latina mother and presents adaptive illness perceptions and help-seeking behaviors (*n*=67) Development: Multiple stakeholders (pharmacist, social work researcher, Fotonovela producer, community members members, clinicians, and professional writers) Provider: *Promotoras* (CHWs) Group vs. Individual: Individual Number/duration of sessions: 1 session, 20–30 min	Exposure to discussion of family communication and intergenerational relationships. Identifies maladaptive communication patterns between immigrant parents and US-born children (*n*=75) Development: Study site's clinicians Provider: Not described Group vs. Individual: Group Number/duration of sessions: 1 session, 45 min	Positive effect: • Increase in mean depression knowledge scores (2.44 vs. 0.02, *p*<0.001) • Decrease in mean antidepressant stigma scores (−1.99 vs. −0.31, *p*=0.002) • Increase in mean self-efficacy scores to identify need for treatment (3.64 vs. 0.13, *p*<0.001) • Higher intent to seek treatment (1.10 vs. 0.70, *p*=0.012). No statistical difference in effect: • Difference in mean pre- to poststigma concern scores (0.03 vs. 0.33, *p*=0.573).
Kieffer (2013)^48^	Design: RCT Population: Latina women, >18 years of age, >20 weeks gestational age (*n*=275) Location: United States Setting: Home and community	Healthy MOMs^*^ curriculum: Spanish language with activities aimed at empowering women during pregnancy and postpartum to develop knowledge and skills to reduce social and environmental barriers to healthy eating and regular exercise (*n*=138) Development: Steering committee of community women and community representatives Provider: Spanish-speaking community residents trained for study Group vs. Individual: Both Number/duration of sessions: 14 sessions, duration n/a	Standard pregnancy education materials about eating and exercise (*n*=137) Development: March of Dimes and the American College of Obstetricians and Gynecologists. Provider: Trained staff from Health MOMs partner Group vs. Individual: Group Number/duration of sessions: 4 sessions/	Positive short-term effect: • Greater decrease in mean CES-D score from baseline to follow-up immediately postintervention (mean difference in change score=−1.83 points; 95% CI: −3.59 to −0.07; *p*=0.042). No statistical difference in long-term effect: • No difference in overall change in mean CES-D score from pretest to post-test between intervention group and control group 95% CI: −3.26 to 0.37, *p*=0.12)
Le (2011)^44^	Design: RCT Population: Latina women aged 18–35 at high risk of depression from Honduras, El Salvador, Guatemala and Mexico (*n*=217) Location: United States Setting: Community and hospital	Culturally tailored, Spanish group psychoeducation intervention (MB course) teaching mood regulation during pregnancy. (*n*=112) Development: Focus groups from staff and clients in community Provider postbachelor trained, bilingual, bicultural staff Group vs. Individual: Group-based Number/duration of sessions 4 sessions×8 weeks, 2 h/session	Usual care—not described (*n*=105)	No statistical difference in effect: • Women in intervention group had lower depression symptoms only immediately postintervention (Cohen's *d*=−0.28, *p*=0.03); • Overall, no significant difference in rates of major depressive episodes (data not shown)
Lee-Lin (2015)^40^	Design: RCT Population: Chinese women (*n*=300) Location: United States Setting: Community-based organization and home	Targeted breast health educational program intervention about breast cancer and mammography screening, using culturally relevant graphics of older and younger Chinese women with Asian landscapes. Including slides, question-and-answer and face-to-face interactions, plus individual counseling (*n*=147) Development: Chinese women focus groups Provider: Trained research staff Group vs. Individual: Both Number/duration of sessions 2 sessions, duration not reported	Chinese version of mammogram information brochure with reminder about follow-up survey (*n*=153) Development: National Cancer Institute Provider: Not described Group vs. Individual: Group Number/duration of sessions: Not described	Positive effect: • Higher rates of mammography completion at 3,6 and 12 months (12 months, 71.4% vs. 42.5%, *p*<0.001) Controlling for age, marital status and age moved to the United States, ORs for mammography completion significantly increased (3 months, OR=8.81, 95% CI 4.83–16.05; 6 months, OR 9.10, 95% CI 3.50–23.62, 12 months, OR 4.61, 95% CI 1.59–13.37)
Lee-Lin (2015)^41^	Design: RCT Population: Chinese women (*n*=300) Location United States Setting: Community-based organization and home	Targeted breast health educational program intervention about breast cancer and mammography screening, using culturally relevant graphics of older and younger Chinese women with Asian landscapes. Including slides, question-and-answer and face-to-face interactions, plus individual counseling (*n*=147) Development: Chinese women focus groups Provider: Trained research staff Group vs. Individual: Both Number/duration of sessions 2 sessions, duration not reported	Chinese version of mammogram information brochure developed by National Cancer Institute with reminder about follow-up survey (*n*=153) Development: National Cancer Institute Provider: Not described Group vs. Individual: Group Number/duration of sessions: Not described	Positive effect: • Higher mean breast cancer knowledge scores at 3 months, 6 months, and 12 months, 5.00 vs. 4.00, *p*=0.03); • Lower mean perceived general barriers by 12 months (2.43 vs. 2.60, *p*=0.01)
Madar (2011)^52^	Design: Cluster RCT Population: Pakistani, Turkish or Somali women, (*n*=40) Location Norway Setting: Child health primary care clinic	Translated and illustrated brochure about importance of vitamin D supplements and sources with infants combined with free vitamin D drops. (*n*=16). Development: Study team Provider: Public health nurses Group vs. Individual: Individual Number/duration of sessions 1 session, duration n/a	Usual care—general information about health issues including recommendation of vitamin D during first visit after delivery with free vitamin D drops (*n*=28) Development: Not described Provider: Public health nurses Group vs. Individual: Individual Number/duration of sessions: 1 session, duration n/a	No statistical difference in effect: • No difference in mean increase of 25-hydroxy vitamin D serum levels (6.3 vs. 2.9, *p*-value not reported.); • No difference in self-reported use of vitamin D (data not shown)
Taylor (2010)^38^	Design: RCT Population: Vietnamese women, 29–79 years’ old (*n*=234) Location: United States Setting: Home	Cervical cancer intervention using translated Vietnamese DVD and pamphlets about importance of Pap testing for all women within the context of Vietnamese beliefs about women's health. (*n*=118) Development: Interviews and focus groups with Vietnamese women Provider: Bilingual, Vietnamese lay health workers Group vs. Individual: Individual Number/duration of sessions 1 session, duration n/a	Mailing of physical activity print materials as well as pedometer with instructions for use—no information on cervical cancer (*n*=116) Development: Not described Provider: Mailing Group vs. Individual: Individual Number/duration of sessions: 1session, duration n/a	No statistical difference in effect: • No difference in rates of Pap test reporting (24% vs. 14%, *p*=0.07) Positive subeffect: • Increased Pap test rates only for women who received Pap test at least once in lifetime (20% vs. 6%, *p*=0.03)
Wu and Lin (2014)^46^	Design: RCT Population: Chinese or Taiwanese women (*n*=193) Location: United States Setting: Home	Interactive, web-based assisted telephone interviewing system delivering cultural counseling messages in English, Mandarin, or Cantonese (*n*=96) Development: Health communication firm and research team plus Advisory Board feedback (medical professionals, cancer survivors, researchers, and community health leaders) Provider: Trained staff with bachelors in health degree Group vs. Individual: Individual Number/duration of sessions: 1 session×60 min	English, mammography pamphlet on breast health explaining procedures and importance of early detection through mammography (*n*=97) Development: National Cancer Institute Provider: Not described Group vs. Individual: Individual Number/duration of sessions: 1 session, duration n/a	No statistical difference in effect: • No difference overall in rates of mammograms at 4-month follow up (40% vs. 33%, *p*=ns). Positive subeffect: • However, women with insurance showed increase mammogram rates (56% vs. 34%, *p*=0.03)

*AMIGAS*, Amigas, Mujeres Latinas, Inform andonos, Gui andonos, y Apoy andonos contra el SIDA; aOR, adjusted odds ratio; BMI, body mass index; CES-D, Center for Epidemiologic Studies-Depression Scale; CHWs, community health workers; CI, confidence interval; HPV, human papillomavirus; n/a, not applicable; ns, non-significant; OR, odds ratio; RCT, randomized control trial.

### Synthesis of results

#### Descriptive analyses

The included studies examined the effects of a range of interventions. All 16 study interventions addressed the cultural and/or linguistic context of the migrant groups studied. Fifteen (94%) combined cultural and linguistic interventions specific to the target migrant population,^37–51^ while one (6%) focused solely on the international migrant women's native language.^52^ Culturally relevant interventions consisted of adapting routine care to address the cultural context of the migrant group (i.e., lifestyle behaviors, ethnic pride, food preferences, cultural roles, images, and/or beliefs) or introducing new, culturally relevant information otherwise not previously available. Linguistically adapted interventions included providing or discussing written or oral information in the international migrant women's native language. The majority (62.5%) of interventions took place, partially or fully, in a community-based locale and/or directly in the client's home setting. The remainder of studies (37.5%) took place in a hospital or primary care clinic. These interventions consisted of 1–16 modules/sessions lasting from 2 min to 6 h per module/session. Interventions were largely (63%) interactive in nature and exclusively targeted individuals or groups (44% and 31%, respectively). Half of the interventions (50%) were delivered by practicing health care professionals and community health workers, while research staff, trained for study purposes, delivered 37.5% of the interventions. Two interventions (12.5%) were delivered solely by a computer.

**FIG. 2.**
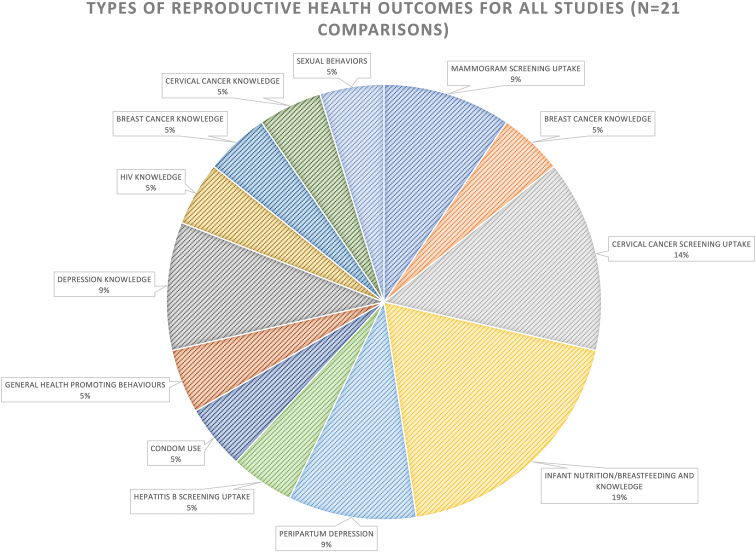
Proportion of types of reproductive health outcomes measured for all studies.

The distribution of intervention effects as being better, worse, mixed, or no different from comparison group outcomes was 88%, 0%, 6%, and 6%, respectively. A wide range of reproductive health-related outcomes were measured in included studies (Fig. 2). After removing studies with a high risk of bias (*n*=5),^37,39,42,45,47^ the remaining studies showed a similar distribution of intervention effects, with 82% reporting better outcomes for international migrant women in the intervention groups than for those in the comparison groups. Of studies included in these analyses, those measuring outcomes directly related to reproductive health included mammogram screening uptake (*n*=3),^40,41,46^ cervical cancer screening uptake (*n*=1),^38^ infant nutrition/breastfeeding knowledge (*n*=2),^49,52^ antepartum/postpartum depression (*n*=2),^44,48^ and condom use (*n*=1).^50^ Studies measuring outcomes indirectly related to reproductive health included general health-promoting behaviors (*n*=1)^51^ and depression knowledge scores (*n*=1).^43^

#### Meta-analyses

Meta-analyses of studies without a high risk of bias (*n*=11) were planned, but only seven studies reported on outcomes similar enough in measurement to permit meta-analyses when grouped by outcome category. These categories included disease prevention activities,^38,40,46,50^ knowledge acquisition,^49,50^ and depression.^44,48^

#### Disease prevention activities

**FIG. 3.**
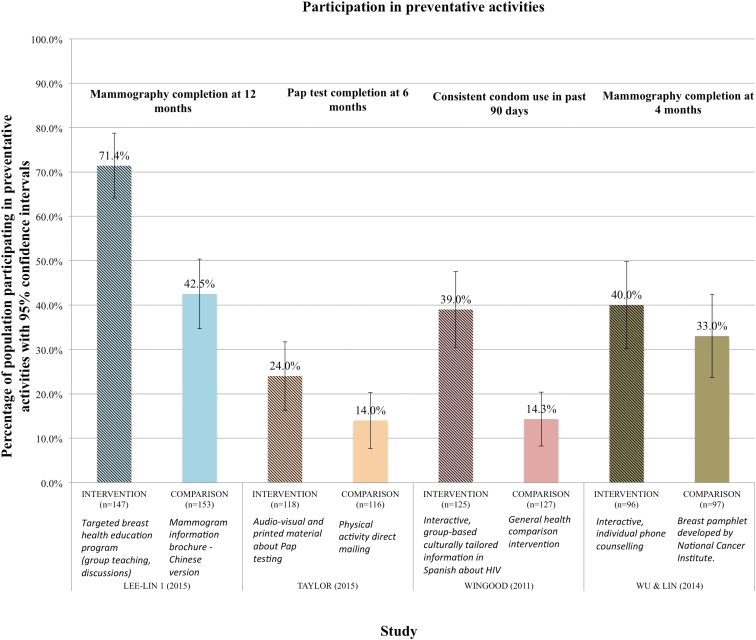
Percentage of population participating in preventative activities by study with 95% CIs. CIs, confidence intervals.

Four studies^38,40,46,50^ reported on interventions used to increase the proportion of international migrant women participating in disease prevention activities (Fig. 3). All four studies took place in the United States and targeted Chinese, Vietnamese, or Latina migrants. All interventions were compared with usual care or an alternative intervention not specific to the cultural or linguistic context of the migrant group. Three interventions used an interactive strategy delivered by trained cultural staff or professional health educators.^40,46,50^ Interventions took place in community and/or home settings and combined the use of audiovisual, computer-interviewing, and printed information through group teaching and/or individual counseling.

**FIG. 4.**
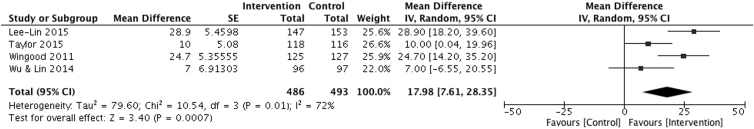
Forest plot for meta-analysis of studies testing effectiveness of interventions on preventative activities.

Together, such interventions demonstrated increased participation in disease prevention activities (mammography screening, Papanicolaou [Pap] test screening, sexually transmitted infections [STI] screening) by almost 18% when compared with usual care or other noncultural or nontranslated interventions (95% CI 7.61–28.35) (Fig. 4).

#### Knowledge acquisition

**FIG. 5.**
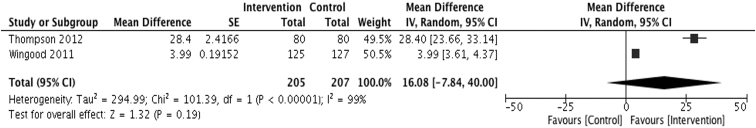
Forest plot for meta-analysis of studies testing effectiveness of interventions on knowledge acquisition.

Two studies measured intervention effects on knowledge acquisition sufficiently enough for meta-analysis.^49,50^ While both interventions addressed the cultural context and language of the Latina migrant groups, only one of them^49^ demonstrated an increase in knowledge with the use of translated interactive computer-based educational modules presented individually in the hospital. Combined, the two interventions were not statistically effective in increasing knowledge among migrant groups (mean difference of 16.08, 95% CI −7.84 to 40.00). The confidence interval is largely positive but wide, suggesting that inadequate statistical power may be responsible for the lack of effect (Fig. 5).

#### Depression

Meta-analysis demonstrated that culturally and linguistically tailored education interventions^44,48^ delivered by research staff had no statistical effect on lowering depression scores for Latinas when compared with usual care or other established informational interventions (data not shown).

### Results of particular aspects of the interventions

#### Site of intervention

Four studies used a community/home-based education intervention program.^40,41,48,51^ Three demonstrated a statistically significant intervention effect, two of which were provided by research staff^40,41^ and one by community health workers.^51^ The fourth intervention delivered by research staff had a positive effect immediately postintervention, but no long-term effect overall.^48^ All four studies offered both group and individual components, but differed in number and duration of sessions, and in methods for providing information (e.g., videos vs. role-playing). Hospital/clinic-based education intervention programs showed mixed results. An eight-session HIV risk-reduction intervention conducted by Latin American government health educators in a health clinic showed an increase in condom use and HIV knowledge,^50^ whereas a four-session behavior therapy intervention conducted by trained research staff in hospital showed no significant long-term effects. Both interventions lasted 2 h or more per session. Altogether, community/home-based educational interventions seem effective, while clinic-based intervention effects are less clear.

#### Delivery system for the intervention

Two studies used computers as the main delivery system for interventions.^46,49^ Compared with those not receiving an intervention, Spanish-language education modules presented on a touch screen increased knowledge scores about infant nutrition.^49^ However, counseling messages delivered to Chinese and Taiwanese women via computer-assisted telephone interviewing systems showed no difference in mammography uptake.^46^ Both interventions were provided in a single session. One study delivered a DVD with a printed brochure in participants' homes to provide information on cervical cancer screening.^38^ However, there was no statistical difference in Pap testing between the intervention and comparison groups. Two studies delivered exclusively printed materials to participants,^43,52^ with mixed results. A translated brochure with illustrations about the importance of vitamin D supplementation showed no statistical difference in vitamin D serum levels between Pakistani, Turkish, and Somali women in the intervention group and those in the usual care group.^52^ A fotonovela, a Spanish comic book, was more effective in increasing mean depression knowledge among Latinas than a discussion of family communication and intergenerational relationships.^43^ Overall, there is no clearly beneficial delivery system for the intervention groups.

### Risk of bias

The risk of bias was assessed for all 16 studies (Tables 2 and 3). For the 13 randomized controlled trials (RCTs), three (23%) were at low risk for bias in all categories, two (15%), were at high risk, and eight (62%) had unclear risk. All three quasiexperimental studies were found to be at high risk of bias. All domains assessed with low risk of bias were associated with blinding of outcome assessment, low attrition, and complete reporting. Unclear risk assessments were associated with sequence generation (i.e., the method of random sequence generation was not described), allocation concealment (i.e., allocation concealment was not described fully or at all), and/or blinding of participants and personnel (i.e., blinding was not described, or provided information was insufficient to assess the effect of blinding).

**Table 2.** Risk-of-Bias Assessment for Randomized-Controlled Trials Using the Cochrane Risk-of-Bias Tool (*n*=13)

Author	Sequence generation (selection bias)	Allocation concealment (selection bias)	Blinding of participants and personnel (performance bias)	Blinding of outcome assessment (detection bias)	Incomplete outcome data (attrition bias)	Selective outcome reporting (reporting bias)	Overall
Koniak-Griffin (2015)^51^	Low	Low	Low	Low	Low	Low	Low
Thompson (2012)^49^	Low	Low	Low	Low	Low	Low	Low
Wingood (2011)^50^	Low	Low	Low	Low	Low	Low	Low
Ma (2015)^37^	Unclear	Unclear	Unclear	Low	Low	High	High
van der veen (2013)^42^	Unclear	Unclear	Low	Unclear	Low	High	High
Hernandez and Organista (2013)^43^	Low	Low	Unclear	Low	Low	Low	Unclear
Kieffer (2013)^48^	Low	Unclear	Low	Low	Low	Low	Unclear
Le Perry and Stuart (2011)^44^	Unclear	Unclear	Unclear	Low	Low	Low	Unclear
Lee-Lin (2015)^40^	Unclear	Unclear	Unclear	Low	Low	Low	Unclear
Lee-Lin (2015)^41^	Unclear	Unclear	Unclear	Low	Low	Low	Unclear
Madar (2011)^52^	Unclear	Unclear	Low	Low	Low	Low	Unclear
Taylor (2010)^38^	Low	Unclear	Low	Low	Low	Low	Unclear
Wu and Lin (2014)^46^	Unclear	Unclear	Low	Low	Low	Low	Unclear

**Table 3.** Risk-of-Bias Assessment for Quasiexperimental Study Trials Using the ROBINS-1 Tool (*n* = 3)

Author	Bias due to confounding	Bias in selection of participants in the study	Bias in classification of interventions	Bias due to deviations from intended interventions	Bias due to missing data	Bias in measurement of outcomes	Bias in selection of the reported results	Overall
Scheinmann (2010)^45^	Low	Low	High (serious)	High (serious)	Low	Low	Low	High (serious)
Wang (2010)^39^	Low	Low	Low	Low	Low	High (serious)	Low	High (serious)
Pitcock (2015)^47^	High (serious)	Low	Low	Low	Low	Low	Low	High (serious)

## Discussion

Our systematic review found that the interventions that most benefited international migrant women's reproductive health were those that adapted to meet the cultural context and/or language skills of the particular migrant group, and focused on disease prevention activities. These included screening for breast and cervical cancer as well as STI screening. Of the four studies meta-analyzed, the two reporting the largest increase in engagement with preventive activity differed in setting, number and duration of sessions, type of intervention provider, and methods used, but shared a multipronged approach, incorporating interactive group dialogue with different forms of media and activities. These approaches included addressing cultural roles, reflecting on cultural norms, presenting information about disease prevention, role-playing, graphics, and poetry. In addition, both interventions were developed by people with close ties to the community. The other two studies, showing less to no significant increase (although still trending toward a positive effect) in preventative activity uptake,^38,46^ involved single-strategy components (i.e., computer-assisted telephone counseling and a DVD with informational pamphlet provided in home). All four interventions also had a community component (i.e., delivered in community clinic, community-based organization, and/or home).

While the importance of overcoming language and cultural barriers in health care for international migrant women is clear, known barriers that challenge migrants' means to achieve positive health outcomes such as payment schemes for health care coverage, health system navigation, perceptions of health and illness, gender roles, and health literacy^53–55^ could impede the effectiveness of culturally and linguistically tailored interventions. For example, one study demonstrated that a culturally tailored intervention was only effective in increasing mammography screening uptake for women covered by health insurance.^46^ Another study showed a beneficial intervention effect only for women who had previously accessed services for Pap testing.^38^

Overall, our findings suggest that interventions to support the uptake of preventive reproductive health activities should focus on culturally and linguistically tailored information, and also incorporate the following: (1) a variety of approaches and materials when providing information; (2) personal exchanges with facilitators in the community; and (3) ways to increase access to care. This is consistent with other systematic reviews showing that multiple-strategy interventions conducted in the community (with face-to-face components, mixed media information, and access-enhancing components) were more effective in increasing uptake of breast and cervical screening among low-income and migrant groups.^56–58^

Our review is largely limited by the number of high-quality reports available for analysis and should be interpreted with caution. Fifty percent of the studies were assessed with an unclear risk of bias, meaning that one or more domains of bias were not reported or could not be ascertained with the information provided. Outcomes combined for meta-analysis were highly heterogeneous, making it difficult to draw convincing conclusions about the effectiveness of different types of interventions. In addition, the true effects of culturally or linguistically adapting interventions could not always be distinguished from the effects of simply providing information or enabling discussions with professionals, health workers, or research staff, because some studies compared providing an intervention with no intervention at all.^45,49^

To our knowledge, this is the first systematic review investigating the effectiveness of interventions supporting the reproductive health of international migrant women in Western-receiving countries. Despite study limitations, our search strategy was broad and inclusive, combining any and all health interventions provided to international migrant women in an effort to identify interventions that could be used or adapted to support their reproductive health. Furthermore, there was no obvious evidence of bias within studies meta-analyzed. Although there exists heterogeneity in the interventions and outcomes reported, we were still able to identify a beneficial effect of culturally and/or linguistically tailored interventions on increasing participation in preventative activities by 18%.

Studies in this review were predominantly conducted in the United States with few reports from countries in Europe, resulting in an emphasis on Latina and Asian international migrant women. Reasons for migration, country of origin, health care systems, and migration policies vary among receiving countries, and can greatly affect the reproductive health outcomes of different migrant groups. This should be taken into consideration when comparing the effects of interventions between international migrant women from different countries of origin and countries of resettlement. Efforts to increase intervention research on different groups of migrants in other Western-receiving countries are strongly recommended.

The results of this review are consistent with the findings of our previous work in that there continues to be a knowledge gap concerning the impact of migration on menopause, contraceptive utilization, abortion care, infertility, HIV/STIs and other infections in pregnancy.^9,10^ In addition, in correlating health outcomes to recommended indicators of migration,^34^ country of birth and ethnicity continue to be predominantly used, while studies continue to inadequately report language fluency, length of time in receiving country, and immigrant status. Failing to address these latter indicators can result in a mis- or underrepresentation of the various ways in which immigration can affect the reproductive health of international migrant women. For example, irregular immigrant status is particularly important, as women may limit their movement in the health care system due to fear of arrest or other negative consequences relating to their status.^6^ Continued and improved reporting on results according to the recommended indicators of migration will serve to help understand the many factors associated with migration and reproductive health outcomes and their importance relative to one another.

Given the dearth of high-quality intervention research aimed at improving the reproductive health of international migrant women, it is imperative that a greater emphasis be given to conducting high-quality intervention studies and that these are fully reported. Until these are published, current evidence suggests that health care professionals should use a variety of approaches and materials specific to preventative reproductive health for international migrant women, favor direct communication between health educators and women, and also work toward facilitating their access to care.

## Supplementary Material

Supplemental data

## Abbreviations Used

*AMIGAS*: Amigas, Mujeres Latinas, Inform andonos, Gui andonos, y Apoy andonos contra el SIDA
aOR: adjusted odds ratio
BMI: body mass index
CES-D: Center for Epidemiologic Studies-Depression Scale
CI: confidence interval
HPV: human papillomavirus
MB: mother and babies
OR: odds ratio
RCT: randomized controlled trial
STI: sexually transmitted infections
WHO: World Health Organization
