# Supplementary material for: Interventions to Support International Migrant Women's Reproductive Health in Western-Receiving Countries: A Systematic Review and Meta-Analysis
Source: Health Equity. 2021 May 25;5(1):356–72. doi: 10.1089/heq.2020.0115 (PMC8170723; doi:10.1089/heq.2020.0115)
Supplement: Supplemental data [file Supp_AppendixSA1.docx]

| **Appendix I. Search Strategy** | |  |  |  |
| --- | --- | --- | --- | --- |
| 1. | (treat* or evaluate* or outcome or intervention or program or teach* or visit* or screen* or referr* or language* or interpret* or translat* or (birth adj plan) or birth-plan or nutrition or support or (social adj marketing) or advoca* or community or counsel* or case-finding or (case adj finding) or case-management or (case adj management) or cultur* or broker or accommodat* or (food adj service) or litera* or communicat* or video* or train* or (picture adj pamphlet) or audio-tape or (audio adj tape) or doula or midwi* or educat* or outreach or safe* or spiritual* or companion* or religio*).mp. |  |  |  |
| 2. | exp Human Migration/ |  |  |  |
| 3. | Refugees/ |  |  |  |
| 4. | (emigra* or immigra* or refugee* or foreigner* or foreign-born or foreign born or migrat* or migrant* or premigra* or newcomer*).ti,ab,kw. |  |  |  |
| 5. | (undocumented or stateless or asylum seek* or detaine*).ti,ab,kw. |  |  |  |
| 6. | (temporary adj2 residen*).ti,ab,kw. |  |  |  |
| 7. | ((human$1 or child* or women) adj2 traffick*).ti,ab,kw. |  |  |  |
| 8. | (unaccompanied adj2 (minor$1 or child*)).ti,ab,kw. |  |  |  |
| 9. | ("forced migrat*" or "unplanned migrat*").ti,ab,kw. |  |  |  |
| 10 | or/2-9 |  |  |  |
| 11 | (australia* or america* or british or english or austria* or canad* or czech or denmark or danish or finland or finnish or france or french or german* or greece or greek* or hungar* or iceland* or ireland or irish or israel*).mp. |  |  |  |
| 12 | (italy or italian* or japan* or korea* or luxemb* or flemish or mexic* or netherlands or dutch or new zealand* or norway or norwegian* or poland or polish or portug* or slovak* or slovenia* or swed* or swiss or switzerland or turkey* or united kingdom or uk or great britain or wales or welsh or scotland or scottish or england or united states or spain or spanish or belg* or chile or chilean or estonia*).mp. |  |  |  |
| 13 | exp North America/ |  |  |  |
| 14 | (bulgaria* or croatia* or cyprus or cypriot* or latvia* or malta or romania* or lithuania*).mp. |  |  |  |
| 15 | exp "Organisation for Economic Co-Operation and Development"/ |  |  |  |
| 16 | OECD.ti,ab. |  |  |  |
| 17 | european union.ti,ab. |  |  |  |
| 18 | or/11-17 |  |  |  |
| 19 | ("differ in" or "difference between" or "differences between" or "differences in" or "different in" or "associated with" or "ethnic differences" or versus or vs or "compared to" or comparing or compare or "compared with" or "association between" or "risk of" or "odds ratio").ti,ab. |  |  |  |
| 20 | 1 and 10 and 18 and 19 |  |  |  |
| 21 | limit 20 to yr="2010 - 2014" |  |  |  |
| 22 | exp animals/ not humans/ |  |  |  |
| 23 | 21 not 22 | | | |
